# Encapsulated Escitalopram and Paroxetine Intranasal Co-Administration: *In Vitro/In Vivo* Evaluation

**DOI:** 10.3389/fphar.2021.751321

**Published:** 2021-12-02

**Authors:** Soraia Silva, Joana Bicker, Carla Fonseca, Nuno R. Ferreira, Carla Vitorino, Gilberto Alves, Amílcar Falcão, Ana Fortuna

**Affiliations:** ^1^ Laboratory of Pharmacology, Faculty of Pharmacy, University of Coimbra, Coimbra, Portugal; ^2^ CIBIT—Coimbra Institute for Biomedical Imaging and Translational Research, University of Coimbra, Coimbra, Portugal; ^3^ Laboratory of Technology, Faculty of Pharmacy, University of Coimbra, Coimbra, Portugal; ^4^ Coimbra Chemistry Center, Department of Chemistry, University of Coimbra, Coimbra, Portugal; ^5^ CICS-UBI—Health Sciences Research Centre, University of Beira Interior, Covilhã, Portugal

**Keywords:** intranasal administration, escitalopram, paroxetine, pharmacokinetics, brain, lungs

## Abstract

Depression is a common mental disorder. Its treatment with selective serotonin reuptake inhibitors (SSRIs) is effective only in a fraction of patients, and pharmacoresistance is increasing steadily. Intranasal (IN) drug delivery to the brain stands out as a promising strategy to improve current therapeutic approaches by operating as a shuttle to overcome the blood–brain barrier. This work aimed to simultaneously administer escitalopram and paroxetine by IN route to mice. For this purpose, three nanostructured lipid carriers (NLC1, NLC2, and BorNLC) and one nanoemulsion (NE) were tested for drug loading. After their characterization, investigation of their impact on nasal cell viability and SSRI permeability assays were performed, using a human nasal RPMI 2650 cell line in air–liquid interface. *In vitro* assays demonstrated that NLCs, including borneol (BorNLC), significantly increased escitalopram permeability (*p* < 0.01) and paroxetine recovery values (*p* < 0.05) in relation to the other formulations and non-encapsulated drugs. IN and intravenous (IV) pharmacokinetic studies performed *in vivo* with a single dose of 2.38 mg/kg demonstrated similar results for escitalopram brain-to-plasma ratios. IN administrations delayed escitalopram peak concentrations in the brain for 15–60 min and no direct nose-to-brain delivery was detected. However, encapsulation with BorNLC considerably decreased escitalopram exposure in the lungs (124 μg min/g) compared with free escitalopram by IN (168 μg min/g) and IV (321 μg min/g) routes. Surprisingly, BorNLC IN instillation increased concentration levels of paroxetine in the brain by five times and accelerated brain drug delivery. Once again, lung exposure was considerably lower with BorNLC (AUC_t_ = 0.433 μg min/g) than that with IV administration (AUC_t_ = 1.01 μg min/g) and non-encapsulated IN formulation (AUC_t_ = 2.82 μg min/g). Direct nose-to-brain delivery was observed for paroxetine IN administration with a direct transport percentage (DTP) of 56.9%. If encapsulated, it increases to 74.2%. These results clearly emphasize that nose-to-brain delivery and lung exposure depend on the formulation and on the characteristics of the drug under investigation. NLCs seem to be an advantageous strategy for nose-to-brain delivery of lipophilic molecules, since they reduce systemic and lung exposure, thereby decreasing adverse effects. For hydrophilic compounds, NLCs are particularly important to decrease lung exposure after IN administration.

## 1 Introduction

Depression is a prevalent mental health disorder and a leading cause of disability, affecting approximately 17% of the global population. It contributes to nearly 800,000 suicides every year and constitutes a major risk factor for the development of neuropsychiatric, cardiovascular, and metabolic disorders, according to the World Health Organization (WHO, 2017). Although multiple treatment strategies are currently applied, including pharmacological treatments, psychotherapies, and various brain stimulation techniques, less than one-half of patients achieve full remission with the first treatment (Akil et al., 2018). In addition, a significant percentage of depressed patients become resistant to available standard treatments (Akil et al., 2018).

Selective serotonin reuptake inhibitors (SSRIs) are first-choice drugs due to their ease of use and more tolerable side effects. Nonetheless, SSRIs exhibit a high potential to interact with other drugs and present an efficacy rate of 70%, encompassing full and partial responses (Pandarakalam, 2018). Several strategies to increase the response of current antidepressants have been clinically applied, such as the use of “combination strategies,” which include two or more antidepressants together (Moret, 2005). However, the adaptation and management of the best combination for a given individual is a challenge. Contrarily to other SSRIs that only exhibit orthosteric activity, paroxetine and escitalopram bind to a secondary allosteric site on the serotonin transporter, leading to a higher inhibition of serotonin reuptake (Mansari et al., 2006). Escitalopram and paroxetine can hence benefit from this combination strategy to promote efficacy and tolerability, by reducing each administered dose (Sanchez et al., 2014).

Currently, there is mounting evidence that ATP-binding efflux transporters, especially P-glycoprotein (P-gp), restrict the uptake of several antidepressants into the brain, thus contributing to the poor success rate of antidepressant therapies (O'Brien et al., 2012; Yu et al., 2018). The access of antidepressants to the biophase may be hampered by the overexpression of P-gp in the apical membrane of enterocytes and in the luminal surface of brain endothelial cells of the blood–brain barrier (BBB). Several studies suggest that escitalopram and paroxetine are P-gp substrates since brain-to-plasma ratios were higher in P-gp knock-out mice than that in the wild type (Uhr et al., 2003; Karlsson et al., 2013; O'Brien et al., 2015; Breitenstein et al., 2016). Paroxetine can also act as a P-gp inhibitor with an *in vitro* half maximal inhibitory concentration (IC_50_) of 10 μg/ml (Feng et al., 2008). However, this value is about 150-fold higher than its therapeutic plasma concentrations (Hiemke et al., 2018), thereby limiting its inhibitory effect *in vivo*. Moreover, both escitalopram and paroxetine are extensively metabolized by enzymes from the cytochrome P450 complex presenting, particularly paroxetine, a significant first-pass metabolism and reduced oral bioavailability (ranging from 30 to 60%). Consequently, both SSRIs exhibit a high potential to develop drug–drug interactions when administered in polytherapy regimen.

Bearing this in mind, optimizing the access of paroxetine and escitalopram to the brain of patients with pharmacoresistant depression is urgently needed. Hence, the intranasal (IN) administration of multiple SSRIs herein investigated, is expected to be an appealing and convenient alternative to conventional oral administration, by avoiding intestinal passage and allowing direct drug delivery to the brain. Indeed, IN administration has revealed to increase drug concentrations at the brain by surpassing the BBB and decreasing the influence of the P-gp–mediated efflux (Gonçalves et al., 2021). Moreover, peripheral systemic exposure and adverse effects decrease, along with the development of drug–drug interactions (Fortuna et al., 2014; Gonçalves et al., 2019; Gonçalves et al., 2020).

Drugs delivered by IN administration can benefit from encapsulation approaches that prevent enzymatic degradation, improve mucoadhesion, nasal permeability, and controlled release (Khan et al., 2017). The use of nanostructured lipid carriers (NLCs) for drug delivery has shown to be a reliable option for IN administration. NLCs are lipid nanoparticles obtained from oil/water emulsions, in which solid and liquid lipids are mixed with an aqueous emulsifier solution (Vitorino et al., 2020). Other advantages of this type of formulation encompass the possibility to incorporate two drugs in the same nanoparticle (co-encapsulation), together with their biocompatibility, biodegradability, and physicochemical stability. Lastly, the reduced costs of production and simple preparation make them good candidates for scale-up manufacturing (Vitorino et al., 2013).

Thus, the goal of the present work was to optimize the IN delivery of escitalopram and paroxetine through a rational *in vitro/in vivo* approach. NLC formulations were initially developed based on our previous work with fluoxetine (Vitorino et al., 2020). Modifications were performed considering the solubility of escitalopram and paroxetine, requiring the test of different lipids to maximize drug loading. Based on these assumptions, borneol inclusion in the NLCs was exploited in an attempt to increase the transport of escitalopram and paroxetine to the brain. Borneol is a compound present in essential oils isolated from plants (Zhang et al., 2021). It improves drug delivery to the central nervous system by increasing BBB permeability (Zhang et al., 2017), promoting drug permeability across the nasal mucosa (Wang et al., 2018), enhancing pinocytotic vesicles, and loosening intercellular tight junctions (Chen and Wang, 2004). Moreover, borneol has antidepressant effects and seems to be a competitive P-gp inhibitor by reducing the efflux of centrally acting drugs that behave as P-gp substrates, such as escitalopram and paroxetine (Chen et al., 2013; Fan et al., 2015).

Herein, the nasal human tumor cell line RPMI 2650 was first used to evaluate the *in vitro* apparent permeability (P_app_) of free and encapsulated drugs across a cell multilayer created at the air–liquid interface, in a semi-porous membrane (Mercier et al., 2018). Thereafter, free and encapsulated drugs with the highest P_app_ were incorporated into a thermoreversible mucoadhesive gel (Gonçalves et al., 2021) to be intranasally administered to mice, in order to evaluate pharmacokinetic parameters in the plasma, lungs, and brain. This gel vehicle was selected due to its potential to promote the direct nose-to-brain delivery of antiepileptic drugs (Serralheiro et al., 2014; Serralheiro et al., 2015; Gonçalves et al., 2019).

## 2 Methods

### 2.1 Chemicals and Reagents

Paroxetine hydrochloride and escitalopram oxalate were kindly donate from Bluepharma (Coimbra, Portugal). Propranolol, used as an internal standard (IS) was obtained from Sigma-Aldrich (St. Louis, MO, United States). Polysorbate 80 (Tween^®^ 80) and borneol were acquired from Sigma-Aldrich, Co. (St. Louis, MO, United States) and Fluka AG. (Buchs SG, Switzerland), respectively. Lauroglycol™ 90 and Precirol^®^ ATO 5 were kindly donated by Gattefossé (Saint-Priest, Cedex, France). Acetonitrile and methanol of high-performance liquid chromatography (HPLC) gradient grade, *n*-hexane, and dimethyl sulfoxide (DMSO) were acquired from Fisher Scientific (Loughborough, United Kingdom). Ultrapure water (HPLC grade, 18.2 MΩ cm) was prepared by means of an Arium^®^ Pro Water System (Sartorius^®^, Goettingen, Germany).

The RPMI 2650 cell line (ATCC^®^ CCL-30™) was obtained from Sigma-Aldrich, and cell culture media and supplements were acquired from Gibco (Thermo Fisher Scientific, United Kingdom). For the preparation of the IN gel, Pluronic F-127 was purchased from Sigma-Aldrich (St. Louis, MO, United States), and Carbopol 974P, from Lubrizol (Wickliffe, OH, United States). Anesthesia was induced with ketamine (Imalgene 1,000^®^, 100 mg/ml) and xylazine (Vetaxilaze 20^®^, 20 mg/ml), both commercially acquired. Sodium chloride 0.9% solution was purchased from B. Braun Medical (Queluz de Baixo, Portugal). Sodium phosphate monobasic dihydrate and orthophosphoric acid, used to prepare 20 mM sodium phosphate buffer (pH = 3.8), were purchased from Merck KGaA (Darmstadt, Germany). All remaining chemicals were obtained from Sigma-Aldrich (St. Louis, MO, United States), unless otherwise stated.

### 2.2 Nanoformulation Characterization

#### 2.2.1 Solubility Studies

Initially, the solubility of escitalopram and paroxetine was determined in triplicate, as described by Vitorino et al. (2020). Briefly, 0.5 g of solid lipid Precirol^®^ ATO 5 was melted in a controlled temperature water bath at 65°C. Escitalopram and paroxetine were added in small amounts until lipid saturation was observed. Regarding liquid lipids (oleic acid, Labrasol^®^, Labrafac™ PG, Lauroglycol™ 90, and Miglyol^®^ 812 N), both drugs were dispersed in screw-capped tubes with the liquid compounds (0.5 ml each) and kept under mechanical stirring for 24 h at 25°C. The samples were then centrifuged at 11,740 g for 5 min using a MiniSpin^®^ (Eppendorf Ibérica S.L., Madrid, Spain). An aliquot of the supernatant was diluted with the mobile phase, filtered through a 0.22-µm membrane, and analyzed.

The quantification of escitalopram and paroxetine was performed through an HPLC method using a Shimadzu HPLC with a diode-array detector (DAD) incorporated into an integrated chromatograph model LC-2040C-3D (Shimadzu Corporation, Tokyo, Japan). The HPLC-DAD apparatus and data acquisition were controlled by using Lab Solutions Software (Shimadzu Corporation, Tokyo, Japan). The chromatographic separation of paroxetine and escitalopram was performed in an 8-min run, using a LiChroCART^®^ Purospher^®^ Star-C18 column (55 × 4 mm; 3 μm particle size from Merck Millipore), maintained at 35°C. The mobile phase was composed of 20 mM phosphate buffer (pH 3.8 adjusted with orthophosphoric acid) and acetonitrile (80:20, v/v) pumped at a flow rate of 1.0 ml/min. A gradient elution program was performed, during which acetonitrile increased to 30% in the first minute and reached 35% in the third minute; after 1 min at 35%, acetonitrile decreased to 20% until the end of the analysis. Detection wavelengths were set at 240 nm for escitalopram and 290 nm for paroxetine. Validation parameters are briefly displayed in Table 1.

**TABLE 1:** Main validation parameters of the HPLC-DAD method applied to quantify escitalopram and paroxetine after *in vitro* permeation studies.

Drug	Mobile phase	Flow rate (ml/min)	Run time (min)	Injection volume	Detection wavelength (nm)	Retention time (min)	Calibration range (µg/ml)	Coefficient of determination (*r* ^2^)	LLOQ (µg/ml)	Precision (% CV)^a^	Accuracy (% Bias)^a^
Escitalopram	20.0 mM phosphate buffer pH 3.80 with orthophosphoric acid/ACN (70.0:30.0, v/v)	1.00	8.00	20.0	240	2.89	0.100–1.00	0.997	0.100	5.01–11.4	−6.62–−4.48
Paroxetine					290	3.56	0.100–2.00	0.996	0.100	9.68–13.7	−11.0–12.2

ACN, acetonitrile; Bias, deviation from nominal value; CV, coefficient of variation; LLOQ, lower limit of quantification.

a Inter-day values (*n* = 3).

#### 2.2.2 Preparation of the Lipid Nanoparticle Dispersions

A hot high-pressure homogenization technique was applied for the production of lipid nanoparticles. Three nanostructured lipid carriers (NLC1, NLC2, and BorNLC) and a nanoemulsion (NE) were initially prepared. The lipid phase (3 g of Lauroglycol™ 90 and Precirol^®^ ATO 5 lipids at different ratios) was heated at a temperature 10°C higher than the melting point of the solid lipid in order to enable a proper solubilization/dispersion of the drug in the lipidic phase and to prevent recrystallization (in the case of NLC) and to facilitate dispersion of the oily phase in the aqueous phase during the homogenization process to obtain a homogeneous formulation. Regarding BorNLC (solid:liquid lipid ratio of 1:3), 50 µl of a borneol solution (50 mg/ml in dichloromethane) was added to the molten lipid phase. Afterward and at the same temperature, 0.270 g of escitalopram and paroxetine were incorporated and emulsified in 30 ml of Tween^®^ 80 (2.5% w/w), with an Ultra-Turrax X1020 (Ystral GmbH, Dottingen, Germany) at 25,000 rpm for 1 min. The obtained pre-emulsion was processed in a preheated EmulsiFlex^®^ C3 (Avestin Inc., Ottawa, Canada) at 1,000 bar for 2.5 min. Finally, the formulations were cooled down for 24 h at 4°C to promote matrix recrystallization and nanoparticle formation.

#### 2.2.3 Characterization of the Lipid Nanoparticles

##### 2.2.3.1 Particle Size and Zeta Potential

The average particle size (PS) and polydispersity index (PdI) were determined by dynamic light scattering, at a 173° detection angle and a temperature of 25.0°C, using the cumulants algorithm. Zeta potential (ZP) was measured by electrophoretic light scattering, at 25.0°C, taking into consideration the Helmholtz–Smoluchowsky equation. The analysis was performed in a Zetasizer Nano ZS (Malvern, Worcestershire, United Kingdom), with samples diluted 100 times with ultrapurified water and measured in triplicate. The results are presented as mean ± standard deviation.

##### 2.2.3.2 Entrapment Efficiency and Drug Loading

The entrapment efficiency (EE) and drug loading (DL) were determined through the measurement of the free drugs present in the aqueous phase of the dispersion. DL is the percentage of the entrapped drug divided by total matrix lipid mass, and it is given by Eq. 1:

$$DL\left(\%\right)=\frac{\left(W_{total\;drug}-W_{free\;drug}\right)}{W_{lipid}}\times 100$$ (1)

where W_total drug_ corresponds to the amount of drug in the nanosystem, W_free drug_ is the drug amount in the aqueous phase, and W_lipid_ is the amount of the lipid phase. EE, which is the amount of drug incorporated into the lipid matrix, was determined according to Eq. 2:

$$EE\left(\%\right)=\frac{\left(W_{total\;drug}-W_{free\;drug}\right)}{W_{total\;drug}}\times 100$$ (2)

To determine the free drug amount for escitalopram and paroxetine, centrifugal ultrafiltration was performed, using centrifugal filter units (Amicon^®^ Ultra 15, Millipore, Germany) with a cut-off at 50 kDa molecular weight. An aliquot of the dispersion (1 ml) was placed in the inner chamber and centrifuged at 4°C for 1 h 30 min at 4,000 g. From the outer chamber of the centrifuge filter unit, the aqueous phase was collected and diluted in the mobile phase (acetonitrile:phosphate buffer pH 3.8, 30:70 v/v) to be then used to quantify the drugs. The total drug amount of both antidepressants was obtained using a specific volume of nanoparticle dispersion, diluted in the mobile phase, and heated for 15 min at 60°C to promote drug extraction from the lipid matrix. Dispersion was centrifuged for 10 min at 11,740 g in a MiniSpin^®^ (Eppendorf Ibérica S.L., Madrid, Spain). All samples were filtered through a 0.22-μm membrane, and drugs were quantified by the HPLC technique described in section 2.2.1.

##### 2.2.3.3 Differential Scanning Calorimetry and X-Ray Diffraction

Analysis of the differential scanning calorimetry (DSC) was obtained using a DSC-204F1 Phoenix differential scanning calorimeter (Netzsch, Germany). The tested lyophilized nanoparticles (2–5 mg) and pure compounds were placed in an aluminum crucible hermetically sealed, and, as reference, an empty crucible was used. For freeze-drying, the aqueous dispersions of NLCs were previously frozen at −80°C for 6 h and then dehydrated under vacuum for 48 h in a Lyph-lock 6 apparatus (Labconco). The samples were submitted to the heating cycles described in Table 2. Parameters such as onset temperature, melting point, and enthalpy were determined using Proteus software (Netzsch, Germany). Pure compounds and lyophilized formulations were analyzed by X-ray diffraction using a MiniFlex 600 X-ray diffractometer (Rigaku, Tokio, Japan), with CuKα radiation at 15 mA and 40 kV. The 2θ scan range was 3–40° with a scan speed of 5 s and a step size of 0.01°.

**TABLE 2:** Heating cycles performed for differential scanning calorimetry (DSC).

Sample	Start temperature (°C)	End temperature (°C)	Heating rate (K/min)	Nitrogen purge flow (ml/min)
Borneol	25.0	220	10.0	20.0
BorNLC		240		
NLC2		170		
Paroxetine		170		
Precirol^®^ ATO 5		70.0		
Escitalopram		190		

##### 2.2.3.4 Attenuated Total Reflectance Fourier Transform Infrared

Attenuated total reflectance Fourier transform infrared (ATR-FTIR) spectra of lyophilized formulations were obtained with a FT-IR/NIR spectrometer (Spectrum 400, Perkin-Elmer, MA, United States) with an ATR accessory fitted with a Zn-Se crystal plate. Samples were placed in the ATR device and measured using 20 scans for each spectrum, with a scan speed of 0.5 cm/s and a resolution of 1 cm^−1^. The spectra were collected between 4,000 and 650 cm^−1^.

#### 2.2.4 Stability Studies

To evaluate the stability of formulations, a LUMiFuge (L.U.M. GmbH, Germany) stability analyzer was used to measure the intensity of transmitted near infrared (NIR) light during sample centrifugation. This analytical centrifugation offers an estimation of instability phenomena. The method enables a rapid and precise means of assessing dispersion stability by measuring separation processes, such as flocculation, coalescence, creaming, and sedimentation.

Using SEPView software v6 (LUM GmbH, Berlin, Germany) for the analysis of transmission profiles, the velocity of separation and instability index were determined. Specifically, the instability index corresponds to the clarification in transmission considering the PS and the separation process at a defined time in the presence of accelerated gravitational force, divided by the maximum clarification. The clarification is the increase in transmission or decrease in particle concentration stemming from the movement of nanoparticles on the bottom of the cell or to the cream layer (Yerramilli and Ghosh, 2017). The values of the instability index range between 0 and 1, where 0 is associated to high stability and 1 indicates high instability. The velocity of separation (µm/s) is calculated from linear regression of the clarification zone and the main instability phenomena detected (creaming or sedimentation) during centrifugation. A high velocity of separation suggests a high instability (ISO, 2013). The formulations were centrifuged at 2,300 g for 50 min at 25°C for this analysis.

### 2.3 *In Vitro* Studies

#### 2.3.1 RPMI 2650 Cell Line

RPMI 2650 (ECACC 88031602) is a human tumor cell line from nasal septum squamous epithelium that was herein selected for viability and permeability experiments. The cells (passage number 20–25) were cultured in Eagle’s minimum essential medium (EMEM, M2279) with 1% non-essential amino acids, 2 mM glutamine, and 1% penicillin–streptomycin mixture, supplemented with 10% heat-inactivated fetal bovine serum. Additionally, the cells were passaged twice a week using 0.25% trypsin–EDTA solution, grown in T75 flasks (Orange Scientific, Braine-l’ Alleud, Belgium), and cultured at 37°C in 5% CO_2_ and 95% relative humidity.

#### 2.3.2 Cell Viability Studies

The influence of the antidepressant drugs and formulations on cell viability was determined by Alamar Blue assay (O'Brien et al., 2000). Briefly, resazurin is metabolically reduced by cells into resorufin, a fluorescent compound that can be quantified. Thus, RPMI 2650 cells were seeded in 96-well plates (Orange Scientific Braine-l’ Alleud, Belgium) at a density of 6 × 10^4^ cells/well and cultured for 24 h. The medium was then removed, and 200 μl of fresh medium (control cells), with 0.5% water (vehicle), 5 mg/ml of free escitalopram and paroxetine, or with each formulation incorporating different drug concentrations, was added to cells and incubated for another 24 h. Afterward, the medium was removed, and a 3-h incubation with 10% Alamar Blue solution in fresh medium (125 mg/ml) was performed. A Biotek Synergy HT microplate reader (Biotek Instruments^®^, Winooski, VT, United States) was used for the fluorescence measurements at 530/590 nm (excitation and emission wavelengths). Eq. 3 was applied for the cell viability calculation:

$$Cell\;\text{v}iability\left(\%\right)=\frac{Fl_{drug}-Fl_{blank}}{Fl_{control}-Fl_{blank}}\times 100$$ (3)

where *Fl*_*drug*_, *Fl*_*blank*_, and *Fl*_*control*_ correspond to the mean fluorescence in wells after incubation with tested conditions, empty wells, and wells without any treatment, respectively. The experiment was performed three times (*n* = 3) with three replicates for each condition. With the obtained results, IC_50_ was calculated for all conditions (ISO, 2009).

#### 2.3.3 Permeation Transport Studies

RPMI 2650 cells were seeded in 12-well polycarbonate microporous Transwell inserts (1.12 cm^2^, 0.4 μm pore size; Corning Costar) at a density of 2 × 10^5^ cells/well. Assays were conducted 23 days after seeding, and the medium from the apical compartment (AP) was removed after 4 days of seeding to induce epithelial differentiation at an air–liquid interface. The culture medium from the basolateral (BL) compartment (0.5 ml) was replaced three times a week. The transepithelial electrical resistance (TEER) of the polarized cell multilayer was monitored with an Evom^®^ STX2 voltohmmeter (WPI, Sarasota, FL, United States) to evaluate membrane integrity. Wells with TEER values higher than 50 Ω cm^2^ were considered adequate for permeation assays (Wengst and Reichl, 2010).

Transport studies were carried out from the apical-to-basolateral (AP-BL) compartment. The culture medium was removed and replaced with Hanks’ balanced salt solution (HBSS) with 10 mM HEPES (pH 7.4). After stabilization at 37°C under shaking (45 rpm), treatment solutions (free drugs or nanoformulations incorporating both escitalopram and paroxetine) were added to the AP side (0.5 ml). Aliquots (240 μl) were removed from the BL side (1.5 ml) after 30, 60, 90, 120, and 180 min of incubation. Replacements with HBSS plus HEPES were performed to avoid hydrostatic pressure gradients. To calculate drug mass balance, aliquots from the AP side at the last time (180 min) were also acquired. Collected samples were injected directly or as described in section 2.2.1 into the chromatographic apparatus to quantify escitalopram and paroxetine by HPLC-DAD.

The P_app_ (in cm/s) was calculated using Eq. 4 (Hubatsch et al., 2007):

$$P_{app}=\frac{\left(dQ/dt\right)}{A\times C_{0}}$$ (4)

where *dQ/dt* is the rate of permeation; *A* is the surface area of the membrane (in cm^2^); and *C*_*0*_ is the initial drug concentration in the AP compartment. For each antidepressant drug, the mass balance was calculated in percentage, according to Eq. 5 (Hellinger et al., 2012):

$$Mass\;balance=\frac{\left(C_{f}^{D}\times V^{D}\right)+\left(C_{f}^{R}\times V^{R}\right)}{C_{0}\times V^{D}}\times 100$$ (5)

where *C*_*f*_ is the final compound concentration in the donor or receiver $\left(C_{f}^{R}\right)$ compartment; *C*_*0*_ is the initial concentration in the donor compartment; and *V* ^*D*^ and *V* ^*R*^ are the volumes of the donor and receiver compartments, respectively.

### 2.4 *In Vivo* Studies

#### 2.4.1 Pharmacokinetic Studies

The purpose of the pharmacokinetic *in vivo* study was to compare the pharmacokinetic profiles of escitalopram and paroxetine in the plasma, brain, and lung tissues after their co-administration by IN (with and without nanoformulation) and intravenous (IV) routes to CD-1 mice.

Healthy adult CD-1 mice (25–30 g) were acquired from Charles River Laboratories (L’Arbresle, France) and accommodated in a relative humidity of 55 ± 5%, a controlled temperature of 20 ± 2°C, and reversed 12 h light–dark cycles. The studies were always performed in the dark phase (active phase between 08h00 and 20h00). A period of at least 7 days was considered for the animals to become familiarized with the environment. Animals had *ad libitum* access to food (4RF21, Mucedola^®^, Italy) and tap water during the entire experiment period.

European Directive (2010/63/EU) regarding the protection of laboratory animals used for scientific purposes, and the Portuguese law on animal welfare (Decree-law 113/2013) were considered throughout all *in vivo* experiments. Moreover, the project was first reviewed and approved by the Animal Welfare Board (ORBEA, 01-2021—Órgão Responsável pelo Bem-Estar Animal) at the Faculty of Pharmacy of the University of Coimbra and the Direção-Geral de Alimentação e Veterinária (DGAV, 0421/000/000/2020, Lisbon, Portugal). All efforts were made to reduce the number of used animals and their suffering.

Before treatment, animals were anesthetized by intraperitoneal administration of a mixture of ketamine (100 mg/kg) and xylazine (10 mg/kg) to keep them immobile during IN or IV administration. Animals were randomly divided into 3 groups (*n* = 28) and subdivided into 7 time-points (*n* = 4). To the first group, free escitalopram and paroxetine were co-administered by the IV route, whereas the second and third groups were intranasally administered with the free or encapsulated drugs, resorting to the nanoformulation selected *in vitro*. While IV administration was performed with an insulin syringe (27 G, 1.0 ml), for IN administration, escitalopram and paroxetine were loaded into a thermoreversible gel prepared as described in Gonçalves et al. (2019). Briefly, Pluronic F-127 (18%, w/v) and Carbopol 974P (0.2%, w/v) were added to cold water, since the first guarantees the thermoreversible characteristic of the nasal gel (Swamy and Abbas, 2012) and the second increases the bioadhesive properties of the gel, thus increasing the residence time in the nasal cavity and improving the central delivery of antidepressant drugs. Both active pharmaceutical ingredients, as free drugs or loaded in a nanoformulation, were incorporated into the gel. Anesthetized animals were instilled with 14–25 µl (2.38 mg/kg) of the gel with a polyurethane tube (24G x 19 mm) attached to a microliter syringe. Formulation delivery was performed into one of the nostrils, where the tube was inserted about 10 mm deep.

Animals were sacrificed by cervical dislocation and decapitation at 5, 15, 30, 60, 120, 240, and 480 min after administration, and heparinized tubes were used for blood collection. Blood samples were centrifuged at 1,252 g for 10 min at 4°C, and plasma supernatants were analyzed by HPLC-DAD. The mice brain and lungs were quickly removed and gently cleaned with a sterile gauze humidified with saline to remove adherent surface blood. Then, these organs were weighed and homogenized in NaCl 0.9% (3 ml/g of tissue) using a tissue homogenizer with a Teflon^®^ pestle from Thomas Scientific (Swedesboro, NJ, United States). Then, tissue homogenates were centrifuged at 1803 g at 4°C for 15 min, and supernatants were analyzed as explained in section 2.4.2.

#### 2.4.2 Quantification of Escitalopram and Paroxetine in Biological Samples

In order to reduce the number of healthy and non-treated animals required for bioanalytical method development and validation, the technique was first optimized and fully validated in human plasma (data not shown). Afterward, it was partially validated in mouse biological samples. The main validation parameters of escitalopram and paroxetine in mice are summarized in Tables 3, 4, in accordance to Bioanalytical Method Validation by the International Council for Harmonization of Technical Requirements for Pharmaceuticals for Human Use (2019) (ICH, 2019).

**TABLE 3:** Main validation parameters of the high-performance liquid chromatography (HPLC) method applied to quantify escitalopram in plasma, brain, and lung matrices.

Validation parameter	Mouse matrices (*n* = 3)		
	Plasma	Brain homogenate	Lung homogenate
Calibration range (µg/ml)	0.0150–1.00	0.00750–0.500	0.0150–1.00
Regression equation^a^	Y = 5.97–0.0335	Y = 13.0–0.0546	Y = 6.51–0.0441
Coefficient of determination (*r* ^2^)^b^	0.996	0.996	0.994
LLOQ (µg/ml)	0.0150	0.00750	0.0150
Inter-day			
Precision (%CV)	4.13–9.83	4.89–12.3	0.836–7.71
Accuracy (%RE)	−9.61 to −1.01	−0.900–1.36	−9.27 to −0.517
Intra-day			
Precision (%CV)	3.85–12.4	5.59–14.0	3.90–14.6
Accuracy (%RE)	−14.3 to −1.47	−9.51 to −1.03	−12.4 to −5.05

LLOQ, lower limit of quantification; CV, coefficient of variation; %RE, deviation from nominal value.

a Equation of the calibration curve is given by the general equation of *y = mx + b*, with *m* corresponding to the slope and *b* to the intercept. The equation represents the peak area signals of escitalopram to that of the internal standard (*y*), versus the corresponding concentration of escitalopram (*x*).

b Weighted linear regression using 1/×^2^ as the best weighting factor.

**TABLE 4:** Main validation parameters of the high-performance liquid chromatography (HPLC) method applied to quantify paroxetine in plasma, brain, and lung matrices.

Validation parameter	Mouse matrices (*n* = 3)		
	Plasma	Brain homogenate	Lung homogenate
Calibration range (µg/ml)	0.0500–2.00	0.0250–1.00	0.0500–2.00
Regression equation^a^	Y = 1.19–0.00671	Y = 1.96 + 0.00428	Y = 1.14–0.0158
Coefficient of determination (*r* ^2^)^b^	0.994	0.996	0.994
LLOQ (µg/ml)	0.0500	0.0250	0.0500
Intra-day			
Precision (%CV)	7.07–14.3	1.25–7.98	10.0–13.4
Accuracy (%RE)	−10.9–0.865	−3.11–13.2	−9.84–9.72
Intra-day			
Precision (%CV)	6.90–12.2	4.97–9.94	5.73–14.6
Accuracy (%RE)	0.974–14.9	−3.31–12.5	−14.7 to −1.71

LLOQ, lower limit of quantification; CV, coefficient of variation; %RE, deviation from nominal value.

a Equation of the calibration curve is given by the general equation of *y = mx + b*, with *m* corresponding to the slope and *b* to the intercept. The equation represents the peak areas signals of paroxetine to that of the internal standard (*y*), versus the corresponding concentration of paroxetine (*x*).

b Weighted linear regression using 1/×^2^ as the best weighting factor.

Escitalopram and paroxetine concentrations were determined in plasma and tissue samples from *in vivo* pharmacokinetic studies applying a liquid–liquid extraction procedure, followed by reversed-phase HPLC-DAD analysis. Briefly, 300 μl of methanol were added to 200 μl of plasma, 200 μl of lung homogenate, or 400 μl of brain homogenate for protein precipitation. After two extractions with 700 μl of *n*-hexane:alcohol isoamyl (98:2, v/v) mixture, organic phases were evaporated. Last, following reconstitution with 100 μl of a mixture of 20 mM phosphate buffer pH 3.8 and acetonitrile (70:30, v/v), the plasma and lung homogenate samples were transferred to a 0.22-μm Costar^®^ Spin-X^®^ centrifugal filter (Corning, Inc., NY, United States) and centrifuged at 12,100 g for 3 min. All final samples were injected (20 µl) into the HPLC-DAD system, in accordance with the analytical conditions described in section 2.2.1. The detection wavelength to quantify the IS was set at 290 nm.

#### 2.4.3 Pharmacokinetic Data Analysis

The maximum concentration (C_max_) values of paroxetine and escitalopram and the corresponding time to reach C_max_ (t_max_) were directly obtained from the experimental data by graphic observation of the mean concentration–time profiles (*n* = 4). The remaining pharmacokinetic parameters were estimated by non-compartmental analysis using WinNonlin^®^ version 6.4 software (Certara, Princeton, NJ). Plasma and tissue concentrations below the lower limit of quantification (LLOQ) of the analytical method were considered zero for the pharmacokinetic data analysis.

For both drugs and in all matrices, the area under the concentration–time curve from time zero (AUC) to the time of the last measurable drug concentration (AUC_t_) was calculated by the linear trapezoidal rule.

To evaluate drug distribution into the brain and lungs after administration, the ratios were determined based on the quotient AUC_t(tissue)_/AUC_t(plasma)_. The drug targeting efficiency (DTE) was calculated following Eq. 6:

$$DTE\left(\%\right)=\frac{\left(AUC_{t\left(brain\right)}/AUC_{t\left(plasma\right)}\right)IN}{\left(AUC_{t\left(brain\right)}/AUC_{t\left(plasma\right)}\right)IV}\times 100$$ (6)

DTE above 100% suggests a higher drug delivery to the central nervous system after IN administration than IV (Fatouh et al., 2017). Finally, to evaluate the direct drug passage from the nose to the brain, the direct transport percentage (DTP) was determined applying Eq. 7:

$$DTP\left(\%\right)=\frac{AUC_{t\left(brain\right)\;IN}-\left[\left(AUC_{t\left(brain\right)\;IV}/AUC_{t\left(plasma\right)\;IV}\right)\times AUC_{t\left(plasma\right)\;IN}\right]}{AUC_{brain\;IN}}\times 100$$ (7)

Values of DTP below 0 indicate better efficiency of the IV route and higher than 0 suggest a direct nose-to-brain transition (Fatouh et al., 2017).

#### 2.4.4 Statistical Data Analysis

Data were processed using GraphPad Prism^®^ 8.4.2 (San Diego, CA, United States). *In vitro* data are expressed as mean ± standard deviation (SD), and *in vivo* pharmacokinetic profiles are expressed as mean ± standard error of the mean (SEM). The Student t-test (paired and unpaired) or one- and two-way analysis of variance (ANOVA) followed by the Tukey posttest were used when appropriate. Differences were considered statistically significant when *p < 0.05, **p < 0.01, and ***p < 0.001.

## 3 Results

### 3.1 Characterization of Nanoformulations

#### 3.1.1 Solubility Studies

Screening the components for the preparation of lipid nanoparticles requires the stepwise selection of solid and liquid lipids or oil. Taking this into account, as well as our previous experience in the development of this type of formulations (Vitorino et al., 2020), the solubility of escitalopram and paroxetine was assessed in the solid and liquid lipids used for the preparation of the lipid nanoparticles. The selection of a glyceride such as Precirol^®^ ATO 5 as the solid lipid with an intermediate melting point (∼56°C) requires lower thermal stress, and it has been biocompatible and acceptable for nose-to-brain delivery (Aboud et al., 2016). In order to maximize loading properties, different liquid lipids were selected to evaluate the solubility of escitalopram and paroxetine (Table 5). Results indicated that Labrasol^®^ displays the highest solubility for escitalopram and paroxetine, followed by Lauroglycol™ 90. Preliminary results showed that NLCs with Labrasol^®^ as liquid lipids presented high values of PS (1,154 ± 43 nm) and PdI (0.672). However, if replaced by Lauroglycol™ 90, PS and PdI decreased to 165 ± 2 nm and 0.273, respectively. Since Lauroglycol™ 90 also presents good solubility for paroxetine and escitalopram and allows the obtainment of smaller particles within a unimodal distribution of PS, it was herein selected as the reference liquid lipid for subsequent tests regarding the optimization of the formulation.

**TABLE 5:** Escitalopram and paroxetine solubility in the tested liquid lipids (*n* = 3, mean ± SD).

Liquid lipid/compound	Escitalopram solubility (mg/ml)	Paroxetine solubility (mg/ml)
Oleic acid	0.840 ± 0.120	1.07 ± 0.580
Labrasol^®^	14.7 ± 14.0	24.0 ± 30.5
Labrafac™ PG	1.34 ± 1.12	n.d.
Lauroglycol™ 90	6.91 ± 0.130	13.8 ± 1.26
Miglyol^®^ 812 N	n.d.	n.d.

n.d., not detected.

#### 3.1.2 Characterization

All formulations were submitted to a first set of tests that included the determination of PS, PdI, ZP, EE, and DL (Table 6). NLC1 clearly evidenced its poorer performance, exhibiting the highest PS and PdI and lowest EE and DL for both escitalopram and paroxetine. On the other hand, NLC2, BorNLC, and NE had comparable performance regarding these parameters. Furthermore, when stored overnight, NLC1 gelled, making it unusable for the purpose of intranasal delivery during *in vivo* studies. Consequently, this formulation was discarded and not tested in the subsequent characterization and *in vitro* screening studies.

**TABLE 6:** Composition of nanostructured lipid carrier (NLC) and nanoemulsion (NE) formulations, respective physicochemical characterization, and evaluation of their performance.

Formulation	Lipid phase		Aqueous phase		Escitalopram paroxetine % (w/w)	Borneol (50 mg/ml)	PS (nm)	PdI	ZP (mV)	EE (%)		DL (%)	
	Precirol^®^ ATO 5% (w/w)	Lauroglycol™ 90% (w/w)	Tween 80% (w/w)	Water %						Escitalopram	Paroxetine	Escitalopram	Paroxetine
NLC1	5.00	5.00	2.50	85.7	0.900	—	546 ± 16.0	0.640	12.0 ± 0.210	17.6 ± 6.86	60.3 ± 9.97	1.01 ± 0.580	3.80 ± 1.42
					0.900								
NLC2	2.50	7.50	2.50	85.7	0.900	—	165 ± 2.01	0.273	11.2 ± 0.400	44.5 ± 5.23	83.1 ± 8.49	4.44 ± 0.700	5.93 ± 1.04
					0.900								
NE	0.00	10.0	2.50	85.7	0.900	—	188 ± 2.10	0.172	17.6 ± 0.890	39.2 ± 7.50	81.1 ± 8.91	2.67 ± 1.58	5.78 ± 2.16
					0.900								
BorNLC	2.50	7.50	2.50	85.7	0.900	50.0 µl	160 ± 2.01	0.273	11.2 ± 0.400	33.9 ± 0.200	78.8 ± 1.06	1.8 ± 0.23	4.00 ± 0.0400
					0.900								

DL, drug loading; EE, entrapment efficiency; PdI, polydispersity index; PS, particle size; ZP, zeta potential.

#### 3.1.3 Additional Structural Aspects

**FIGURE 1:**
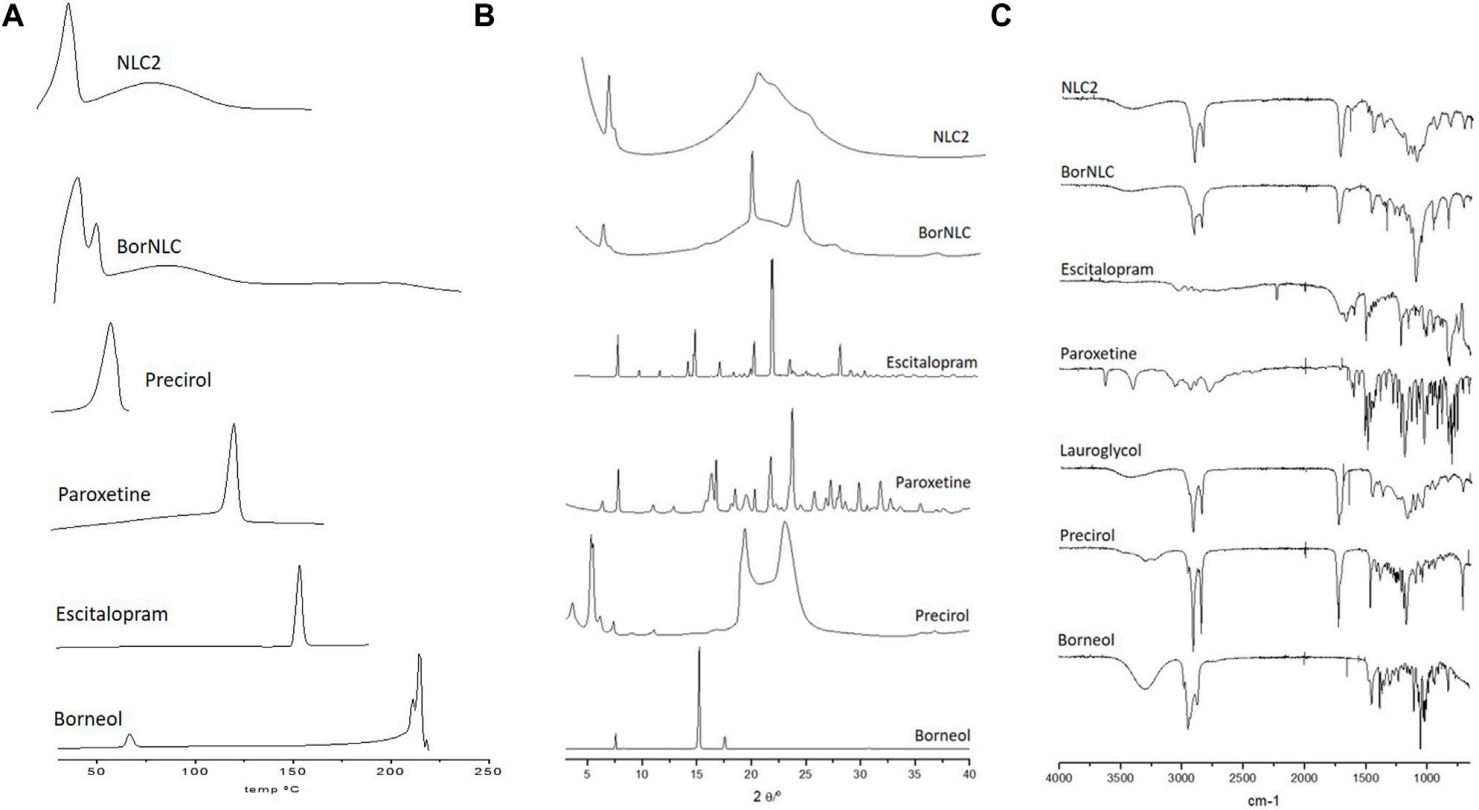
DSC thermograms **(A)**, X-ray diffractograms **(B),** and ATR-FTIR spectra **(C)** for pure compounds (Precirol^®^ ATO 5, escitalopram oxalate, paroxetine hydrochloride, and borneol) and loading formulations NCL2 and BorNCL. The liquid lipid Lauryglycol™ 90 was evaluated in ATR-FTIR spectra since only solid samples are permitted for XRD analysis.

DSC curves of paroxetine, escitalopram, and NLC formulations were herein performed, and the results are displayed in Figure 1A. Precirol^®^ ATO 5 exhibits a melting peak at 52.4°C, and DSC formulation curves of both NLC2 and BorNLC show similar peaks, despite having remained smaller and broader after particle preparation. The reason may be associated with the high aspect ratio of particles that increases surface energy. This creates an energetically suboptimal state, resulting in a reduction of the melting point (Makled et al., 2017). A similar behavior seems to occur regarding borneol that presented a melting point of 213°C, while BorNLC revealed a smaller and broader peak around that same temperature. The DSC thermogram of paroxetine and escitalopram showed only one distinct endothermic peak at 118°C and 150.7°C, respectively, corresponding to their melting temperature (Pina et al., 2014; Pinto et al., 2018). The absence of this thermal transition in the loaded formulations confirms the molecular dispersion in the lipid matrix.

The data obtained from DSC curves are similar with the results observed in XRD spectral analysis (Figure 1B). The spectra obtained for paroxetine are similar to what is presented in the literature for isoform I, and the typical peaks (2Ɵ of 24.10, 22.08, 30.12, 16.64, 17.04, 28.35, 32.11, 27.51, 18.75, and 26.05) (Sugi et al., 2000) are not visible in the XRD spectral analysis of the NLCs. Escitalopram followed the same pattern, where none of the characteristic peaks were observed in the NLCs. Instead, diffractograms essentially exhibited crystalline aspects attributed to the presence of the solid lipid, Precirol^®^ ATO 5.

To complement the information acquired from DSC and X-Ray diffraction, ATR-FTIR was performed to evaluate the intermolecular interactions in the formulations (Figure 1C). The Precirol^®^ ATO 5 spectrum demonstrates three important peaks: 1736.79 cm^−1^ (C=O stretch), 1729.60 cm^−1^ and 1,472.43 cm^−1^ (C=C stretching), and 2,913.57 and 2,849.06 cm^−1^ (C–H stretching) (Kumbhar and Pokharkar, 2013). The main absorption peaks of Lauroglycol™ 90 occurred at similar wavelengths (1736.77 cm^−1^ for C=O stretch, 1,458.44 cm^−1^ for C=C stretching, and 2,922.85 and 2,853.57 cm^−1^ for C–H stretching) probably because it presents the same type of bonds than Precirol^®^ ATO 5. Nonetheless, the higher complexity of the Precirol^®^ ATO 5 spectra reflects its bigger molecular size than that with Lauroglycol™ 90. Both absorption peaks indicate the molecular signature of the NLCs spectra. It was also possible to observe that the IR spectrum of pure paroxetine and escitalopram has different peaks when compared with the NLCs. Absorption peaks from the active pharmaceutical ingredients were not found in FTIR spectra of NCLs, similar to DSC and XRD results (Figure 1).

#### 3.1.4 Stability Testing

Stability assessment of each formulation is a key issue to ensure product quality. This implies the control of phase separation, sedimentation, or creaming that affect safety and efficacy (Pathak et al., 2018). The use of analytical centrifugation under an accelerated gravitational field as the stability test allows a rapid comparison of formulation shelf-life instead of waiting long-time at Earth gravitation.

**FIGURE 2:**
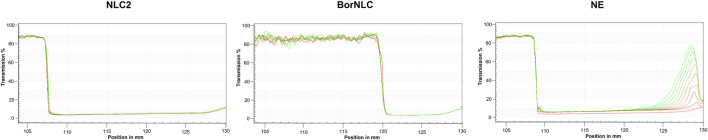
Physical stability of the formulations using analytical centrifugation to predict potential destabilization processes. **(A)** Instability indices of NLC2, BorNLC, and NE. **(B)** Transmission profiles of NLC2, BorNLC, and NE formulations elucidating the impact of the liquid:solid lipid ratio and the incorporation of borneol.

Considering the results obtained from the instability index, NE displays the highest instability index (0.25) compared to other formulations and a separation profile characteristic of a creaming formation, corroborating the previous results already reported (Vitorino et al., 2020). Conversely, formulations containing solid lipids, such as NLC2 and BorNLC, exhibited lower instability index values (<0.05), suggesting their good stability. Interestingly, the incorporation of borneol in the formulation further increased its stability. In both NLCs, no observable separation process was noted during the test period (Figure 2). As expected, NE had the highest velocity of separation (4,693 μm/s) compared with NLC2 (47.70 μm/s) and BorNLC (not detected), corroborating its high instability. The good stability of NLC2 and BorNLC is confirmed by the low velocity of separation, especially in the presence of borneol. Following this set of studies, NE was discarded from the additional structural tests.

### 3.2 *In Vitro* Cell Viability and Permeability Studies

Cell viability assessed through the Alamar Blue assay allowed the determination of IC_50_ for escitalopram and paroxetine (Table 7). Lower IC_50_ values were found for nanoformulations in relation to free drugs, evidencing their higher impact on cell viability. In particular, NE and BorNLC were the most toxic formulations, but no statistical differences were found between BorNLC and NLC2. Cytotoxicity differences between treated and untreated cells were determined in order to define the maximal tested concentration that did not compromise cell viability (Table 7). Accordingly, NE compromised cell viability in the lowest tested drug concentrations. Hence, permeation assays were carried out with the concentrations achieved in Table 7 and 5 μg/ml for free drugs.

**TABLE 7:** Half inhibitory concentrations (IC_50_) and maximal tested concentration without loss of cell viability of free and encapsulated escitalopram and paroxetine in the RPMI 2650 cell line for 24 h (n = 3).

Formulations	IC_50_ (µg/ml)		Maximal tested concentration without loss of cell viability (µg/ml)	
	Escitalopram	Paroxetine	Escitalopram	Paroxetine
Free drugs	12.8 ± 0.730	12.8 ± 0.730	8.33	8.33
NLC2	5.33 ± 0.260	7.12 ± 0.350	4.45	5.93
BorNLC	2.12 ± 0.230	4.71 ± 0.500	1.80	4.00
NE	2.34 ± 0.240	5.06 ± 0.520	1.48	3.21

NE, nanoemulsion; NLC, nanostructured lipid carrier.

**FIGURE 3:**
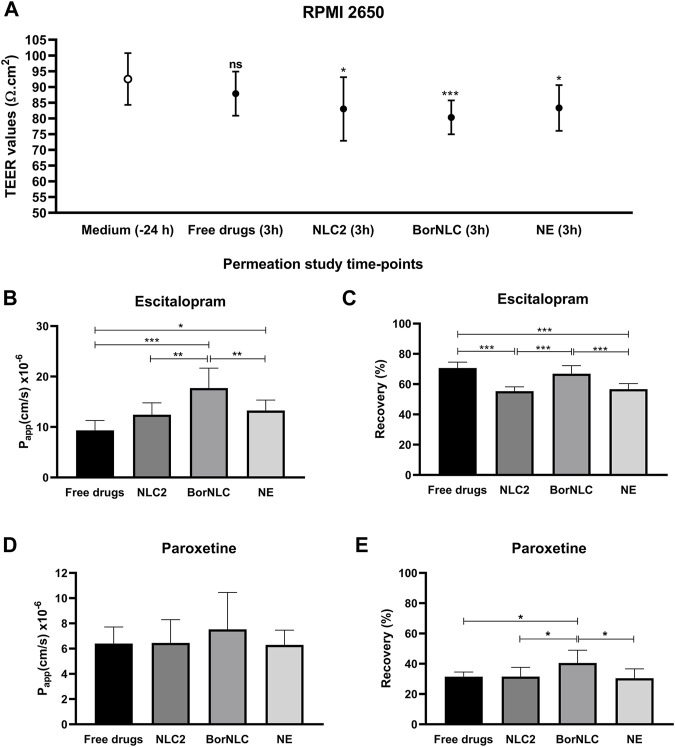
Transepithelial electrical resistance (TEER) values of the RPMI 2650 cell line, measured in the EMEM medium or Hanks’ balanced salt solution (HBSS) with 10 mM HEPES (pH 7.4) with treatment conditions [free escitalopram and paroxetine (free drugs), NLC2, BorNLC, or NE], after the permeation assay. The paired student t-test was performed in comparison with the values obtained 24 h before the assay. NS, not significant, *p < 0.05 and ***p < 0.001. **(A)** Escitalopram apparent permeability coefficient (P_app_) **(B)** and recovery values **(C)**; paroxetine P_app_ **(D)** and recovery values **(E)** after a 3-h incubation with each treatment. One-way analysis of variance (ANOVA) with multiple comparison test was performed, *p < 0.05, **p < 0.01, and ***p < 0.001. All data are presented as mean ± standard deviation (*n* = 3 in triplicate).

Permeation studies were performed 23 days after seeding RPMI 2650 cells, with constant measurement of TEER to ensure the presence of sufficiently restrictive tight junctions. On the day before experiments, TEER values were 92.5 ± 8.21 Ω cm^2^, and in the end of permeation studies, TEER was measured to determine if nanoformulations could affect the formation of the tight junctions (Figure 3A). Regarding free drugs, TEER values were close to the initial values obtained the previous day (87.7 ± 6.93 Ω cm^2^) with no significant differences (*p* = 0.108). However, cells incubated with nanoformulations decreased TEER values, reaching 82.9 ± 10.1 Ω cm^2^ (*p* = 0.013) and 83.4 ± 7.15 Ω cm^2^ (*p* = 0.028) for NLC2 and NE, respectively. Cells incubated with BorNLC showed significantly lower TEER values with 80.3 ± 6.93 Ω cm^2^ (*p* < 0.001) than the values measured the day before (Figure 3A).

The P_app_ and recovery values obtained for escitalopram and paroxetine are presented in Figures 3B–E. Regarding escitalopram, the highest P_app_ value (17.7 × 10^−6^ ± 3.95 × 10^−6^ cm/s) was obtained for BorNLC (Figure 3B), while the lowest was observed for the free drug (9.34 × 10^−6^ ± 1.94 × 10^−6^ cm/s) followed by NLC2 and NE (12.5 × 10^−6^ ± 2.34 × 10^−6^ cm/s and 13.3 × 10^−6^ ± 2.07 × 10^−6^ cm/s, respectively). NE and BorNLC significantly increased escitalopram permeability compared with the free drug (*p* < 0.05), in contrast to NLC2. Also, statistically significant differences were found between BorNLC and the other formulations (*p* < 0.01). Regarding paroxetine, although no statistically significant differences were found between treatment conditions, the highest recovery values and P_app_ were attained with BorNLC (7.52 × 10^−6^ ± 2.94 × 10^−6^ cm/s) (Figures 3D,E).

Based on these results, BorNLC was selected to be intranasally administered to mice, in order to describe escitalopram and paroxetine pharmacokinetics and compare them with free drug administration by IV route.

### 3.3 *In vivo* Pharmacokinetic Analysis

**FIGURE 4:**
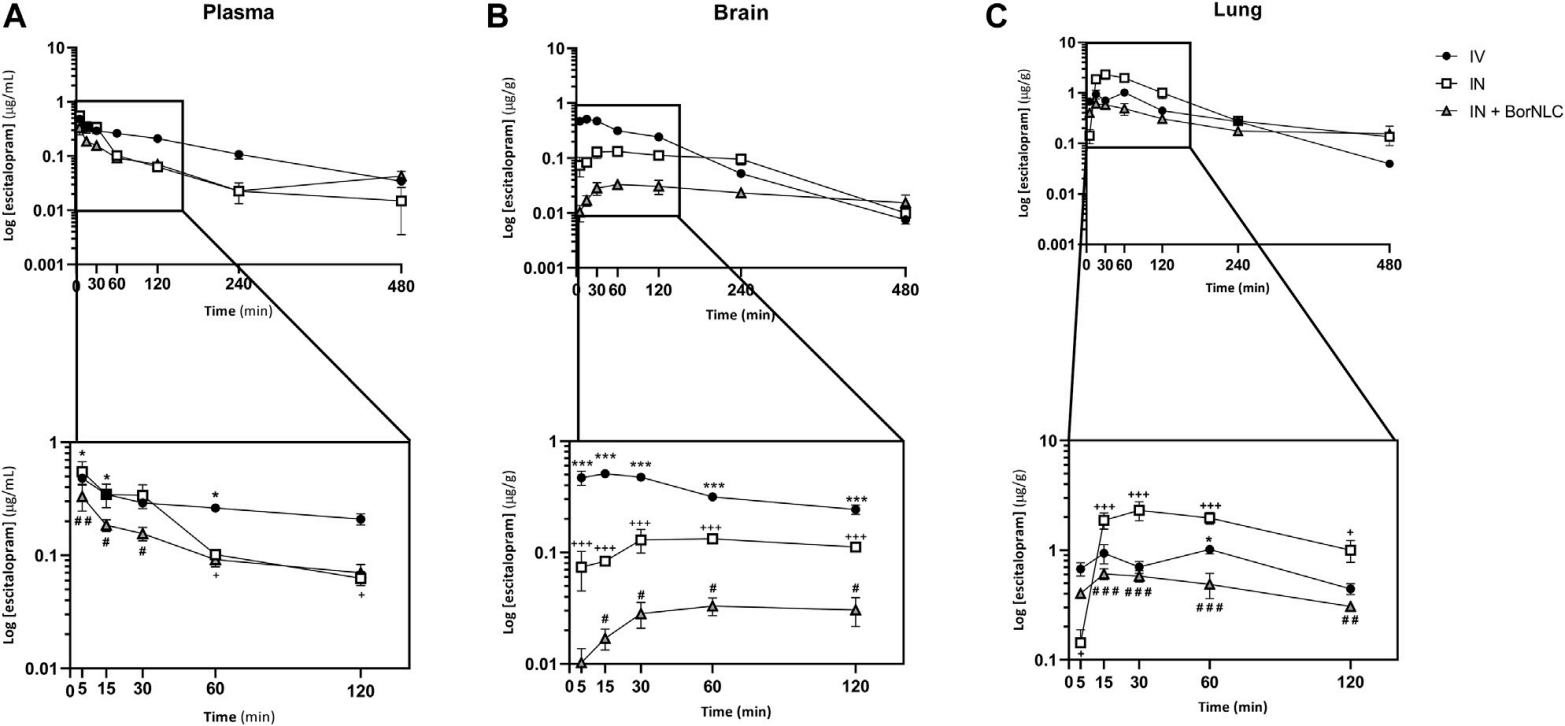
Concentration–time profiles of escitalopram up to 480 min after dosing in the plasma **(A)**, brain **(B),** and lungs **(C)** following intravenous (IV), intranasal (IN), and IN with BorNLC (IN + BorNLC) co-administration with paroxetine (2.38 mg/kg) to mice. Symbols represent the mean values ± SEM (*n* = 4). *p < 0.05, **p < 0.01, and ***p < 0.001. * represents differences between IV vs. IN + BorNLC, ^+^between IV vs. IN, and ^#^ between IN vs. IN + BorNLC.

**FIGURE 5:**
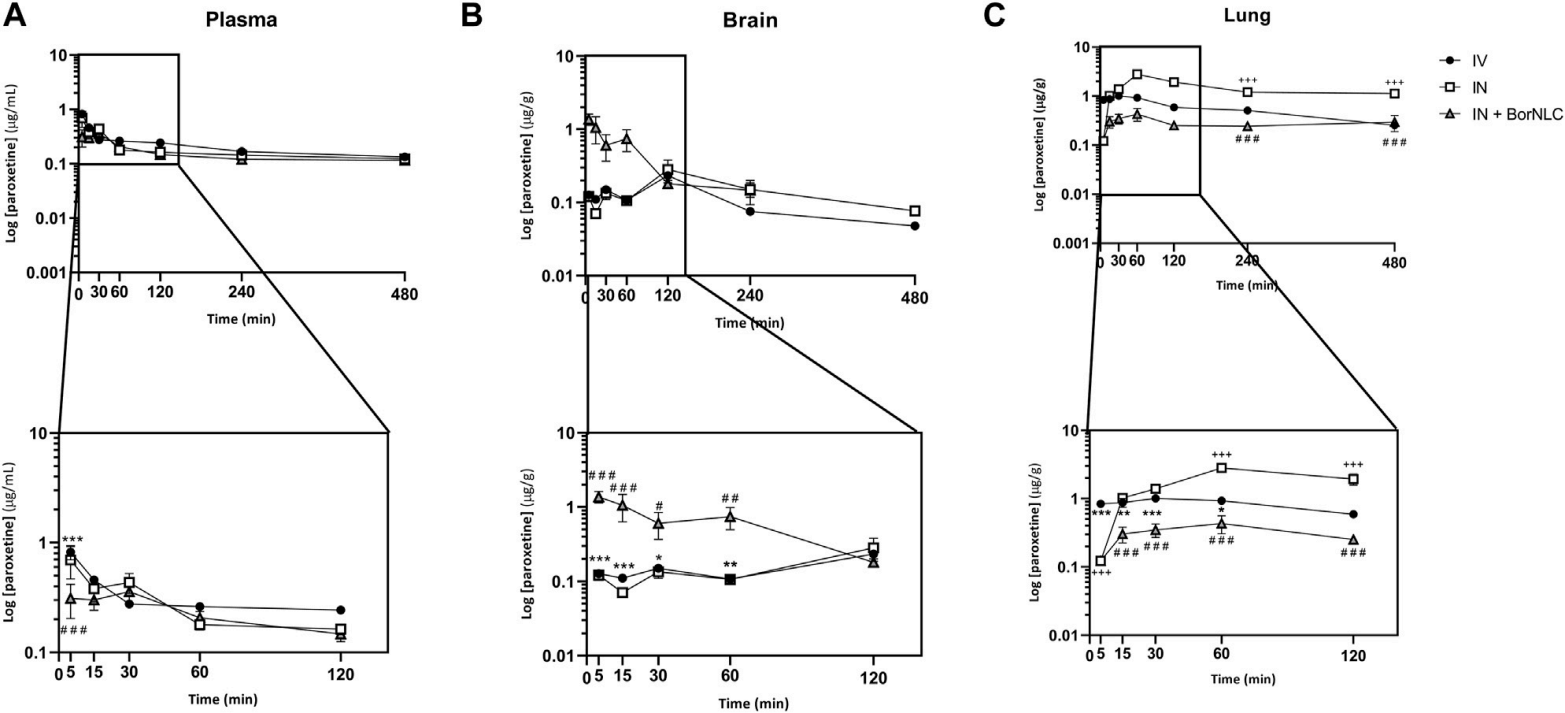
Concentration-time profiles of paroxetine up to 480 min after dosing in the plasma **(A)**, brain **(B),** and lungs **(C)** following intravenous (IV), intranasal (IN), and IN with BorNLC (IN + BorNLC) co-administration with escitalopram (2.38 mg/kg) to mice. Symbols represent the mean values ± SEM (*n* = 4). *p < 0.05, **p < 0.01, and ***p < 0.001. * represents differences between IV vs. IN + BorNLC, ^+^between IV vs. IN, and ^#^ between IN vs. IN + BorNLC.

The mean plasma, brain, and lung concentration–time profiles of escitalopram are shown in Figure 4. The corresponding pharmacokinetic parameters obtained after IV and IN administration of the free antidepressant and IN administration of BorNLC to mice are shown in Table 8. The same information obtained for paroxetine is displayed in Figure 5 and Table 9. No information comprising IN administration of paroxetine or escitalopram was found in the literature. Kreilgaard et al. measured plasma concentrations for 6.00 h after a single subcutaneous injection of escitalopram (0.240, 1.00, and 3.90 mg/kg) or paroxetine (0.270, 1.00, and 4.40 mg/kg) (Kreilgaard et al., 2008). The authors observed a rapid absorption for escitalopram and paroxetine based on semi-log concentration–time profiles and t_max_ values in the plasma (3.00–5.40 min for escitalopram; 4.80–5.40 min for paroxetine). These results were identical to those evidenced in this study for free paroxetine and escitalopram (5.00 min) (Tables 8 and 9).

**TABLE 8:** Pharmacokinetic parameters of escitalopram in plasma, brain, and lung tissues following their intravenous (IV) and intranasal (IN) free or encapsulated co-administration with paroxetine to mice. The administered dose was 2.38 mg/kg.

Escitalopram pharmacokinetic parameter^a^	Plasma			Brain			Lung		
	IV	IN	IN + BorNLC	IV	IN	IN + BorNLC	IV	IN	IN + BorNLC
t_max_ (min)	5.00	5.00	5.00	15.0	60.0	60.0	60.0	30.0	15.0
C_max_ (µg/ml)	0.482	0.550	0.384	0.511^b^	0.133^b^	0.0285^b^	1.02^b^	2.31^b^	0.608^b^
AUC_t_ (µg min/ml)	68.7	30.4	28.3	59.8^c^	26.2^c^	6.25^c^	168^c^	321^c^	124^c^
AUC_tbrain_/AUC_tplasma_				0.871	0.860	0.221			
AUC_tlung_/AUC_tplasma_							2.45	10.6	4.37
DTE (%)					98.8	25.4			
DTP (%)					−1.21	−294			

a Parameters were estimated using the mean concentration–time profiles obtained from four different animals per time-point (*n* = 4).

b Values expressed in µg/g.

c Values expressed in µg.min/g; AUC_t_, area under the concentration time-curve from time zero to the last quantifiable drug concentration; C_max_, maximum peak concentration; DTE, drug targeting efficiency; DTP, direct transport percentage; IN, intranasal; IV, intravenous; NLC, nanostructured lipid carrier; t_max_, time to achieve the maximum peak concentration.

**TABLE 9:** Pharmacokinetic parameters of paroxetine in plasma, brain, and lung tissues following their intravenous (IV) and intranasal (IN) free or encapsulated co-administration with escitalopram to mice. The administered dose was 2.38 mg/kg.

Paroxetine pharmacokinetic parameter^a^	Plasma			Brain			Lung		
	IV	IN	IN + BorNLC	IV	IN	IN + BorNLC	IV	IN	IN + BorNLC
t_max_ (min)	5.00	5.00	30.0	120	120	5.00	30.0	60.0	60.0
C_max_ (µg/ml)	0.820	0.696	0.359	0.233^b^	0.283^b^	1.37^b^	1.01^b^	2.82^b^	0.433^b^
AUC_t_ (µg min/ml)	98.0	83.2	68.8	34.6^c^	68.2^c^	94.3^c^	257^c^	699^c^	134^c^
AUC_tbrain_/AUC_tplasma_				0.354	0.821	1.37			
AUC_tlung_/AUC_tplasma_							2.63	8.40	1.94
DTE (%)					232	388			
DTP (%)					56.9	74.2			

a Parameters were estimated using the mean concentration–time profiles obtained from four different animals per time-point (*n* = 4).

b Values expressed in µg/g.

c Values expressed in µg min/g; AUC_t_, area under the concentration time-curve from time zero to the last quantifiable drug concentration; C_max_, maximum peak concentration; DTE, drug targeting efficiency; DTP, direct transport percentage; IN, intranasal; IV, intravenous; NLC, nanostructured lipid carrier, t_max_, time to achieve the maximum peak concentration.

Considering Figure 4, escitalopram plasma concentrations were lower after IN administration of BorNLC formulation, indicating statistical differences when compared with free escitalopram administration by IV and IN routes (*p* < 0.05) (Figure 4A). All three administrations presented the same t_max_ (5.00 min), but IN administration led to AUC_t(plasma)_ values less than 50% of those obtained after IV injection (28.3–30.4 vs. 68.7 μg min/ml). When administered as BorNLC formulation, escitalopram showed the lowest C_max_ (0.384 μg/ml) (Table 8), suggesting a slower and scarce systemic absorption.

**FIGURE 6:**
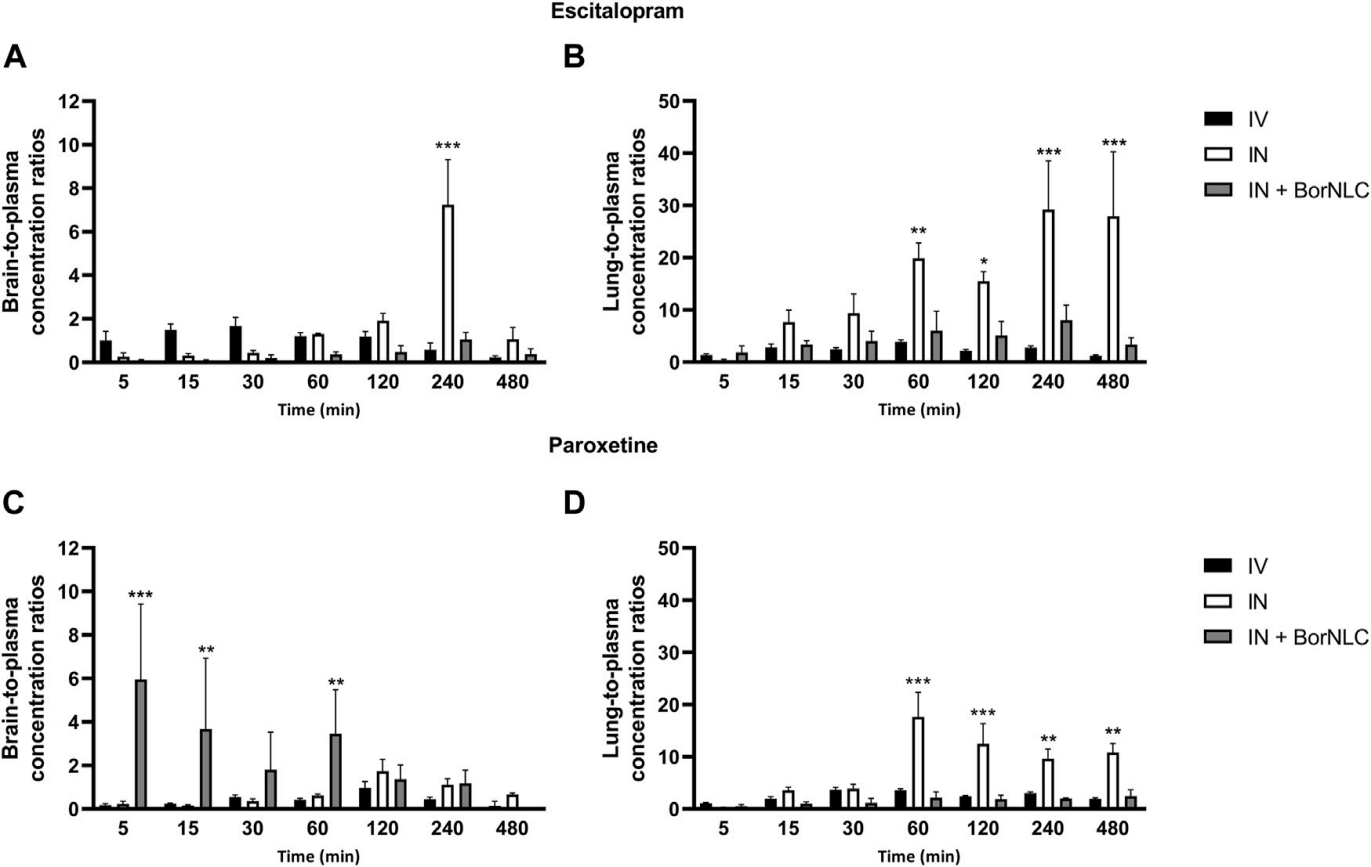
Tissue-to-plasma concentration ratios of escitalopram **(A,B)** and paroxetine **(C,D)** in the brain and lungs up to 480 min after intravenous (IV), intranasal (IN), and IN delivery of BorNLC (IN + BorNLC) co-administration (2.38 mg/kg) to mice. Two-way analysis of variance (ANOVA) with multiple comparison test was performed, *p < 0.05, **p < 0.01, and ***p < 0.001.

A similar pattern was observed regarding brain concentration–time profiles. Between 15.0 and 120 min, IV injection revealed the highest concentrations for escitalopram (*p* < 0.001), followed by free drug IN administration and BorNLC (*p* < 0.05) (Figure 4B). Both IN administrations attained C_max_ later than the IV injection (60.0 vs. 15.0 min), but C_max_ and AUC_t(brain)_ were 80.0% higher after free drug IN instillation than BorNLC. Therefore, these results evidence that borneol did not improve drug exposure in the brain (Table 8) and are corroborated by the superior DTE and absolute DTP obtained for free IN escitalopram (98.8% and −1.21%, respectively) in relation to the BorNLC formulation (25.4% and -294%, respectively, Table 8). Indeed, in a study performed by Jacobsen et al., escitalopram was administered to mice by the intraperitoneal route in doses of 0.25, 0.5, 1, or 2 mg/kg (Jacobsen et al., 2014). Escitalopram levels were determined in the plasma and brain at 30.0, 60.0, and 90.0 min post- dosing. Interestingly, clearance in the brain was slower than that in the plasma, as shown by increasing brain-to-plasma ratios (Jacobsen et al., 2014). Despite different dosing routes, these observations were in accordance with the results of the present study, for the IN route, given by brain-to-plasma ratios increasing until 240 min (Figure 6A).

In Figure 4C, the highest lung concentrations of escitalopram were found after IN free drug administration (*p* < 0.013). Lung concentrations after IV administration were statistically higher than those observed after IN instillation only in the first 5.00 min (*p* = 0.027). Remarkably, when encapsulated, escitalopram displayed the lowest concentration values in the lungs (with the exception of 5.00 min). This is the same tendency as already aforementioned for the plasma and brain. According to Table 8, C_max_ was attained earlier in the lungs than in the brain when intranasally administered. Moreover, after free drug IN administration, C_max_ was 2.3 and 3.8 times superior than those observed after IV and BorNLC IN administration, respectively. All three animal groups exhibited AUC_t(lung)_/AUC_t(plasma)_ ratios for escitalopram higher than 1, suggesting a high preference for the lungs. As observed in Figure 6B, lung-to-plasma ratios significantly increase after 60.0 min for IN free escitalopram administration.

Interestingly, paroxetine demonstrated marked differences in relation to the pharmacokinetic profiles of escitalopram. For instance, in the plasma, concentrations were comparable between the three formulations. Indeed, the lowest paroxetine concentrations were found 5.00 min after its IN administration as BorNLC (*p* < 0.001), and no more statistically significant differences were found (Figure 5A). Nonetheless, the similarities among plasma concentrations (Table 9) demonstrate that the BorNLC IN administration achieved t_max_ later (30.0 min vs. 5.00 min for the other two administrations), and C_max_ decreased approximately 48–56%. Second, IN administration of BorNLC led to higher brain concentrations than IV and IN up to 60 min post-dosing (*p* < 0.011, Figure 5B). This behavior is completely distinct from that of escitalopram. As summarized in Table 9, brain C_max_ of paroxetine after BorNLC IN administration was attained earlier (5 min vs. 120 min), and it was more than 4 times higher than IV and IN free drug administrations. IN administration of BorNLC enabled a 63% higher brain exposure (given by AUC_t_) than that of IV injection, while free paroxetine IN administration only increased drug exposure by 49%. IN BorNLC significantly increased brain-to-plasma ratios in the first 60.0 min post-administration (Figure 6C). The total AUC_t(brain)_/AUC_t(plasma)_ ratio for the IV route was 0.35, suggesting paroxetine preference to plasma, but this ratio increased to 0.82 and 1.37 after IN free drug or BorNLC administrations, respectively (Table 9). Since DTE values after IN administration were above 100%, a higher drug delivery to the brain was obtained than using IV. Moreover, the achieved DTP indicates that more than 50% of paroxetine suffers nose-to-brain transport. DTE and DTP were higher for BorNLC than that for free drug IN administration (Table 9). These findings undoubtedly emphasize that the encapsulation of paroxetine in BorNLC not only increases brain exposure but also its direct nose-to-brain delivery.

Our previous experience in IN administration (Gonçalves et al., 2019) has demonstrated that drugs attain the lungs at high concentrations. On the other hand, antidepressants such as SSRIs are associated with respiratory depression adverse effects. Therefore, determination of drug exposure in the lungs becomes essential to infer whether the NLCs could protect the lungs and avoid side effects. The lung concentration–time profiles of paroxetine demonstrate that the highest concentrations were found after IN free drug administration (*p* < 0.043), particularly from 30.0 to 480 min (Figure 5C). Importantly, C_max_ was 6.5 times inferior after drug encapsulation, identical to AUC_t(lung)_, which was 5 times lower with IN BorNLC than that with free drug. Similar to IN free escitalopram, significantly higher lung-to-plasma ratios were found after 60.0 min for IN free paroxetine, but not with IN BorNLC (Figure 6D). These results provide evidence that paroxetine incorporation into BorNLCs protects the lungs by reducing drug exposure, as it had been previously observed for escitalopram. This is particularly relevant because antidepressant drugs have associated with respiratory complications (Rosenberg et al., 2017; Masarwa et al., 2019).

## 4 Conclusion

Herein, a rational approach encompassing an *in vitro* permeability assay across RPMI 2650 cells, followed by an *in vivo* biodisposition study, was applied to screen antidepressant drugs and assess their distribution into the brain. This study revealed the pharmacokinetic parameters of escitalopram and paroxetine after IN co-administration applying a thermoreversible gel. The influence of encapsulation with BorNLC was also evaluated.

*In vivo* results revealed the potential of the IN route for the delivery of antidepressants to the central nervous system, particularly for lipophilic compounds (e.g., paroxetine). The pharmacokinetic parameters of free escitalopram obtained after IN administration were similar to those of IV injection, and no direct nose-to-brain transport was detected. Encapsulation of escitalopram decreased drug exposure in all matrices. Therefore, the incorporation of escitalopram into BorNLC did not appear to provide a better pharmacokinetic profile than that of the IV route. In contrast, IN administration increased paroxetine exposure in the brain, which was considerably higher after encapsulation. Paroxetine demonstrated a direct nose-to-brain delivery and good target efficiency, which were significantly improved after encapsulation in BorNLC.

This study emphasizes the importance of the physical and chemical properties of the drug when exploiting IN administration and encapsulation to promote direct brain access. Specifically, lipophilic compounds are more likely to benefit from IN dosing and encapsulation than hydrophilic drugs. Particularly BorNLC seems to be a good strategy because, in one hand, it decreases drug toxicity in cell lines and, on the other hand, it seems to increase nose-to-brain delivery. This is shown by a higher brain exposure and direct nose-to-brain delivery and lower systemic and lung exposure. Moreover, it was also evident that lung exposure to both antidepressants decreased after their encapsulation, which may potentially contribute to the reduction of adverse effects in the lungs after IN administration. Therefore, IN administration of antidepressants is expected to be a relevant tool for the treatment of depression.

## Data Availability

The original contributions presented in the study are included in the article/Supplementary Material, further inquiries can be directed to the corresponding authors.

## Nomenclature

AP: apical
ATR-FTIR: attenuated total reflectance Fourier transform infrared
AUC: area under the curve
BBB: blood–brain barrier
BL: basolateral
C_max_: maximum concentration
DAD: diode array detector
DL: drug loading
DMSO: dimethyl sulfoxide
DSC: differential scanning calorimetry
DTE: drug targeting efficiency
DTP: direct transport percentage
EE: entrapment efficiency
EMEM: Eagle’s minimum essential medium
HBSS: Hanks’ balanced salt solution
HPLC: high-performance liquid chromatography
IC_50_: half maximal inhibitory concentration
IN: intranasal
IS: internal standard
IV: intravenous
LLOQ: lower limit of quantification
NE: nanoemulsion
NLCs: nanostructured lipid carriers
P_app_: apparent permeability
PdI: polydispersity index
P-gp: P-glycoprotein
PS: particle size
SSRIs: selective serotonin reuptake inhibitors
TEER: transepithelial electrical resistance
t_max_: time of maximum concentration
ZP: zeta potential.
